# Retraction: Chitosan/xanthan gum based hydrogels as potential carrier for an antiviral drug: fabrication, characterization, and safety evaluation

**DOI:** 10.3389/fchem.2026.1945269

**Published:** 2026-07-28

**Authors:** Frontiers Editorial Office

The journal retracts the 4 February 2020 article cited above.

Following publication, concerns were raised regarding the integrity of figures in this article. This included concerns regarding image duplication in the original article and, subsequently, concerns regarding the corrected material published in the related corrigendum. In the course of the investigation, further discrepancies were identified between the published figures and the files later supplied by the authors.

As the issues could not be sufficiently addressed by the authors, the editorial office no longer has confidence in the reliability of the images reported in the article. The original article is therefore retracted.

This retraction was approved by the Chief Editor of *Frontiers in Chemistry* and the Chief Executive Editor of Frontiers. The authors agreed to this retraction. We would like to thank Dr. Elisabeth Bik, who brought this case to the attention of the Editorial Office.

